# Rapid Detection of Multi-Resistance Strains Carrying *mcr*-1 Gene Using Recombinase-Aided Amplification Directly on Clinical Samples

**DOI:** 10.3389/fmicb.2022.852488

**Published:** 2022-03-31

**Authors:** Zheng Fan, Yanling Feng, Wenjian Xu, Junxia Feng, Chao Yan, Tongtong Fu, Hanqing Zhao, Jinghua Cui, Lin Gan, Shiyu Liu, Shuheng Du, Rui Zhang, Ziying Xu, Nannan Li, Guanhua Xue, Jing Yuan

**Affiliations:** ^1^Department of Bacteriology, Capital Institute of Pediatrics, Beijing, China; ^2^Children’s Hospital Affiliated to Capital Institute of Pediatrics, Beijing, China

**Keywords:** RAA assay, *mcr*-1, *E. coli*, colistin, children

## Abstract

With the increasingly severe problem of bacterial resistance, colistin, as the last line of defense, has attracted attention again. Mobile colistin resistance (*mcr*-1) gene is involved in the horizontal transmission of colistin resistance in Gram-negative bacteria (GNB), which is a serious threat to human health. Therefore, rapid detection of *mcr*-1 gene presence in clinical samples is crucial. In this study, a Recombinase-aided amplification(RAA) method for *mcr*-1 was successfully constructed, with sensitivity of 20 copies/reaction. In addition, amplification signal could only be detected in the strain containing *mcr*-1 gene among 14 different bacterial species. The method was then used to test a total of 672 clinical samples from a pediatric hospital in Beijing. Five strains harbored *mcr*-1 genes were isolated from *mcr*-1-positive clinical samples and identified as *Escherichia coli*. Multi-locus sequence typing (MLST) analysis showed that the five *E. coli* belonged to different ST types. Notably, the *mcr*-1 gene from the isolates could be transferred conjugately to the recipient strain *E. coli* J53, with highest transfer efficiency up to 57–58%, suggesting that the *mcr*-1 gene was located on the plasmid. These findings showed that the RAA assay has potential to be a rapid and sensitive *mcr*-1 gene screening test for clinical samples, and *mcr*-1 could be transmitted vertically and horizontally between and within bacterial species in a plasmid-mediated manner.

## Introduction

For the past few decades, polymyxins are used for the treatment of multidrug-resistant GNB when better treatment options are not available (Li et al., 2006; Lim et al., 2010). The main mechanism of action of polymyxin is that its positive charge binds to the negative charge of the phosphate groups of lipid A on lipopolysaccharide (LPS) localized on the outer membrane of Gram-negative bacteria (GNB) (Schindler and Osborn, 1979; Srimal et al., 1996; Moffatt et al., 2019). Ca^2+^ is essential for maintaining the structural stability of LPS, and polymyxin can replace LPS-bound Mg^2+^ and Ca^2+^, increasing the permeability of bacterial outer membrane and causing bacterial death (Schindler and Osborn, 1979). Many factors, such as two-component systems PhoP/PhoQ (Elizabeth et al., 2021) and efflux pumps MexXY-OprM (Poole et al., 2015), can influence the resistance of bacteria to polymyxin. However, the plasmid-mediated *mcr* genes can rapidly spread *via* horizontal gene transfer (HGT) among humans, animals, and the environment, posing the greatest risk to human health (Hussein et al., 2021).

The *mcr* genes encode phosphoethanolamine (pEtN) transferase enzymes, which can reduce the electrostatic interaction between polymyxin and lipid A of LPS by binding pEtN moiety to the lipid A of GNB, creating bacteria resistant to polymyxin (Baron et al., 2016). So far, ten variants of the *mcr* gene, *mcr*-1 to *mcr*-10, have been identified in various bacteria (Wang et al., 2020). The plasmid-mediated colistin resistance gene *mcr*-1 was the first to be identified from *E. coli* in 2015 and is more widely disseminated than the other nine variants, while *mcr*-2 to *mcr*-10 have only occasionally been reported (Liu et al., 2016; Xiaomin et al., 2020). The spread of the *mcr*-1 gene makes multidrug-resistant GNB more resistant to polymyxins, which poses a serious threat to public health (Xiaomin et al., 2020). Hence, a rapid and accurate method of detecting the *mcr*-1 gene carrier strain would be helpful to guide clinical medication and inhibit the *mcr*-1 gene spread.

The polymerase chain reaction (PCR) method has been used to detect the presence of *mcr* genes, but it is time consuming (Rebelo et al., 2018; Mentasti et al., 2021). RAA assay is a highly efficient method for the rapid detection of specific target genes. Based on isothermal amplification technology, the RAA assay can be completed within 15–30 min at 39°C and has been widely used in clinical applications, such as for identifying New Delhi Metallo-β-Lactamase Gene (Feng et al., 2021), *bla*_KPC_ (Zhang et al., 2021) and other applications (Fan et al., 2019; Qi et al., 2019; Shen et al., 2019; Xue et al., 2020a,b). Here, an RAA assay was developed to detect the *mcr*-1 gene in clinical samples, which was proven to have high specificity and sensitivity. To further analyze the characteristics of strains harboring *mcr*-1 genes obtained from clinical samples, the minimum inhibitory concentrations (MICs) and the HGT of *mcr*-1 to these isolates were investigated.

## Materials and Methods

### Bacterial Strains, Growth Conditions, and Primers

Information on all of the strains used in this study is listed in Table 1. All strains were cultured in Luria-Bertani (LB) broth (5 g/L yeast extract, 10 g/L sodium chloride, and 10 g/L tryptone) at 37°C in a shaker at 200 rpm. All primers involved in the construction of plasmid, PCR and RAA assay are listed in Table 2.

**TABLE 1:** Bacterial strains used in this study.

Bacterial strain	Description/function	Source
*E. coli*^mcr^** ^–1^	Specificity of the RAA Assay	Our microorganism center
*Klebsiella pneumoniae* 2,146	Specificity of the RAA Assay	Our microorganism center
*E. coli* ATCC 25922	Specificity of the RAA Assay	Our microorganism center
*Shigella sonnei*	Specificity of the RAA Assay	Our microorganism center
*Salmonella enteritidis*	Specificity of the RAA Assay	Our microorganism center
*Acinetobacter baumannii*	Specificity of the RAA Assay	Clinical isolate
*Klebsiella oxytoca*	Specificity of the RAA Assay	Clinical isolate
*Enterobacter aerogenes*	Specificity of the RAA Assay	Clinical isolate
*Proteus mirabilis*	Specificity of the RAA Assay	Clinical isolate
*Enterobacter cloacae*	Specificity of the RAA Assay	Clinical isolate
*Serratia marcescens*	Specificity of the RAA Assay	Clinical isolate
*Campylobacter jejuni*	Specificity of the RAA Assay	Clinical isolate
*Citrobacter freundii*	Specificity of the RAA Assay	Clinical isolate
*Pseudomonas aeruginosa*	Specificity of the RAA Assay	Clinical isolate
*E. coli*/*mcr-*1 1	*mcr*-1 positive strain 1	Clinical isolate
*E. coli*/*mcr-*1 2	*mcr*-1 positive strain 2	Clinical isolate
*E. coli*/*mcr-*1 3	*mcr*-1 positive strain 3	Clinical isolate
*E. coli*/*mcr-*1 4	*mcr*-1 positive strain 4	Clinical isolate
*E. coli*/*mcr-*1 5	*mcr*-1 positive strain 5	Clinical isolate
*E. coli* J53	Recipient strain	Our microorganism center
*E. coli*-J53TCp*E. coli*/*mcr*-1 1	*E. coli*-J53 contains *mcr*-1 which was transferred from *E. coli*/*mcr*-1 1	This study
*E. coli*-J53TCp*E. coli*/*mcr*-1 2	*E. coli*-J53 contains *mcr*-1 which was transferred from *E. coli*/*mcr*-1 2	This study
*E. coli*-J53TCp*E. coli*/*mcr*-1 3	*E. coli*-J53 contains *mcr*-1 which was transferred from *E. coli*/*mcr*-1 3	This study
*E. coli*-J53TCp*E. coli*/*mcr*-1 4	*E. coli*-J53 contains *mcr*-1 which was transferred from *E. coli*/*mcr*-1 4	This study
*E. coli*-J53TCp*E. coli*/*mcr*-1 5	*E. coli*-J53 contains *mcr*-1 which was transferred from *E. coli*/*mcr*-1 5	This study

**TABLE 2:** Primers used in this study.

Primer	Sequence (5’→3’)	Function
*mcr*-1-F1	ATGATGCAGCATACTTCTGT	Plasmid construction
*mcr*-1-R1	TCAGCGGATGAATGCGGTGC	Plasmid construction
*mcr*-1-F	CGTTCAGCAGTCATTATGCCAGTTTCTTTCGCGTGC	RAA assay
*mcr*-1-R	CTTACGCATATCAGGCTTGGTTGCTTGTACCGC	RAA assay
*mcr*-1-P	GCCAATCTACTCGGTGGGTAAGCTTGCCAG[FAM-dT][THF][BHQ-dT]TGAGTATAAAAAAGC3’-block	RAA assay
16S-F	TGGAGCATGTGGTTTAATTC GATGCAACGC	RAA assay
16S-R	GGATAAGGGTTGCGCTCGTT GCGGGACTTAA	RAA assay
16S-P	TGACATCCACAGAACTTTCCAGAGATGGATTGG[FAM-dT]G[THF]C[BHQ-dT] TCGGGAACTGTGAGAC [30 -block]	RAA Assay
dinBoF	GTTTTCCCAGTCACGACGTTGTATGAGAGGTGAGCAATGCGTA	MLST
dinB2oR	TTGTGAGCGGATAACAATTTCCGTAGCCCCATCGCTTCCAG	MLST
icd2oF	GTTTTCCCAGTCACGACGTTGTAATTCGCTTCCCGGAACATTG	MLST
icdoR	TTGTGAGCGGATAACAATTTCATGATCGCGTCACCAAAYTC	MLST
pabB2oF	GTTTTCCCAGTCACGACGTTGTAAATCCAATATGACCCGCGAG	MLST
pabBoR	TTGTGAGCGGATAACAATTTCGGTTCCAGTTCGTCGATAAT	MLST
polB2oF	GTTTTCCCAGTCACGACGTTGTAGGCGGCTATGTGATGGATTC	MLST
polBoR	TTGTGAGCGGATAACAATTTCGGTTGGCATCAGAAAACGGC	MLST
putP2oF	GTTTTCCCAGTCACGACGTTGTACTGTTTAACCCGTGGATTGC	MLST
putPoR	TTGTGAGCGGATAACAATTTCGCATCGGCCTCGGCAAAGCG	MLST
trpAoF	GTTTTCCCAGTCACGACGTTGTAGCTACGAATCTCTGTTTGCC	MLST
trpAoR	TTGTGAGCGGATAACAATTTCGCTTTCATCGGTTGTACAAA	MLST
trpB2oF	GTTTTCCCAGTCACGACGTTGTACACTATATGCTGGGCACCGC	MLST
trpBoR	TTGTGAGCGGATAACAATTTCCCTCGTGCTTTCAAAATATC	MLST
uidAoF	GTTTTCCCAGTCACGACGTTGTACATTACGGCAAAGTGTGGGTCAAT	MLST
uidAoR	TTGTGAGCGGATAACAATTTCCCATCAGCACGTTATCGAATCCTT	MLST

### Acquisition, Isolation, and Identification of Clinical Strains

Six hundred and seventy-two stool samples were collected from inpatients of Capital Institute of Pediatrics, Beijing, China. After dilution, stool samples with different dilution gradients were plated on LB plates with 2 mg/L of colistin sulfate salt and incubated at 35–37°C for 24 h. The screened single colonies were identified using the VITEK^®^ 2 compact system (bioMérieux, Nürtingen, Germany). Standard PCR and RAA assay were used simultaneously to detect whether the strains harbored the *mcr*-1 gene. Meanwhile, a sample of approximately 200 mg was subjected to DNA extraction using a kit (Vazyme Biotech Co., Ltd., Nanjing, China) for further use.

### Primer Design for the Recombinase-Aided Amplification Assay

The sequence of the *mcr*-1 gene was downloaded from the National Center for Biotechnology Information (NCBI) GenBank database (NCBI Reference Sequence: NG_050417.1). The primers and probes were manually designed under the principles of RAA primer and probe. Briefly, the primer size was between 30 and 35 bp, the probe size was between 46 and 52 bp and the final product size was between 100 and 200 bp. The specificity of primers and probes was confirmed by NCBI primer-specific BLAST analysis and the hairpins and primer dimers were analyzed by Primer Primier 5. As an internal positive control, the primers and the probe of the 16S rRNA gene were designed in its conserved region. Related primers and probes involved in this study were synthesized by Sangon Biotech (Shanghai, China) and purified by high-performance liquid chromatography.

### Analytical Sensitivity and Specificity of the Recombinase-Aided Amplification Assay

The full-length *mcr*-1 gene was amplified by PCR and cloned into vector pUC57 (Tiangen Biotech Co., Ltd., Beijing, China) by TA cloning, and the recombinant plasmid was called pUC57-*mcr*-1. The analytical sensitivity of the RAA assay was determined using 10-fold serial dilutions of the recombinant plasmid pUC57-*mcr*-1 ranging from 10^7^ to 10^0^ copies/μL. The analytical specificity of the RAA assay was evaluated by amplifying the *mcr*-1 and 16S rRNA genes from 14 different strains, respectively *E. coli*^mcr^**^–1^, *Klebsiella pneumoniae* 2146, *E. coli* ATCC 25922, *Pseudomonas aeruginosa* ATCC 27853, *Shigella sonnei*, *Salmonella enteritidis*, *Acinetobacter baumannii*, *K. oxytoca*, *Enterobacter aerogenes*, *Proteus mirabilis*, *Enterobacter cloacae*, *Serratia marcescens*, *Campylobacter jejuni*, and *Citrobacter freundii*. The *E. coli*^mcr^**^–1^ was used as a positive control and sterile water was used as a negative control.

### Recombinase-Aided Amplification Assay

A commercial RAA kit (Jiangsu Qitian Bio-Tech Co., Ltd., China) was used for the RAA assays. The RAA assays were performed as described previously (Feng et al., 2021). Briefly, a 50 μL reaction mixture was prepared first, which was made of reaction buffer (25 μL), DNase-free water (15.7 μL), 10 μM primer F (2.1 μL), 10 μM primer R (2.1 μL), DNA template (2 μL), 280 mM magnesium acetate (2.5 μL), and 10 μM probe (0.6 μL). Then, the reaction mixture was added to a tube with the lyophilized form of RAA enzyme mix and the tube was mixed briefly and incubated for 4 min in a B6100 Oscillation mixer (QT-RAA-B6100; Jiangsu Qitian Bio-Tech Co., Ltd., China). Finally, a fluorescence detector (QT-RAA-1620; Jiangsu Qitian Bio-Tech Co., Ltd.) was used to measure the fluorescence for 20 min at 39°C.

### Standard Polymerase Chain Reaction Assay

To detect *mcr*-1 gene, PCR was performed in a 20 μL reaction mix containing the following: 10 μL of PCR Master Mix reagent (Tiangen Biotech Co., Ltd., Beijing, China), 1 μL of 10 μM *mcr*-1-F primer (5′-CGTTCAGCAGTCATTATGCCAGTTTCTTTCGCGTGC-3′) and *mcr*-1-R primer (5′-CTTACGCATATCAGGCTTGGT TGCTTGTACCGC-3′), 1 μL of DNA template and 7 μL of double-distilled water. The PCR cycling conditions were 95°C for 3 min, followed by 35 cycles at 95°C for 30 s, 58°C for 30 s, and 72°C for 1 min. The final extension step was 72°C for 15 min. The PCR products were sent to Sangon Biotech for sequencing.

### Multi-Locus Sequence Typing Analysis

All *mcr*-1-positive *E. coli* isolates were identified using the VITEK^®^ 2 compact system and screened in accordance with the protocols presented on the MLST website.^1^ Eight housekeeping genes, namely, *dinB* (DNA polymerase), *icdA* (isocitrate dehydrogenase), *pabB* (p-aminobenzoate synthase), *polB* (polymerase PolII), *putP* (proline permease), *trpA* (tryptophan synthase subunit A), *trpB* (tryptophan synthase subunit B), and *uidA* (beta-glucuronidase), were detected.

### Antimicrobials Susceptibility Testing

To analyze the characteristics of strains harboring *mcr*-1 genes, MICs were mainly determined using a VITEK^®^ 2 system (bioMérieux, Nürtingen, Germany). *Escherichia coli* ATCC25922 was used for quality control. The 2020 Clinical Laboratory Standards Institute’s threshold was used to as reference. In addition, the MICs of colistin and polymyxin B were determined by the twofold serial dilution method, as previously described (Fan et al., 2021). Briefly, the strains harboring *mcr*-1 genes were grown in LB broth at 37°C until the optical density at 600 nm (OD600) reached 1.0. Next, 100 μL of the bacterial suspension (5 × 10^5^ CFU/mL) and different concentrations of diluted antibiotics were added to each well of a 96-well plate (Corning). The 96-well plate was incubated without agitation at 37°C for 24 h. The minimum antibiotic concentration to visibly inhibit bacterial growth was recorded as the MIC.

### Horizontal Gene Transfer Assay

To detect the HGT of *mcr*-1, colistin-resistant isolates served as donor strains, with sodium azide-resistant *E. coli* J53 as recipient. Briefly, 500 μL of each donor strain and 500 μL of the recipient in LB broth were mixed, centrifuged at 8,000 rpm, and the bacterial precipitates were resuspended on 50 μL of LB broth. The resuspended bacteria were then added to round filter papers pre-placed on nutritional agar plates and cultured overnight at 37°C. Transconjugant bacteria were selected on LB plates containing sodium azide (100 mg/mL) and colistin (4 μg/mL). Meanwhile, the same number of bacteria were plated on LB plates only containing sodium azide (100 mg/mL). The number of bacterial cells was determined by serial dilution and plating. The transfer efficiency was calculated by dividing the number of successfully transformed strains by the total number of receptor strains.

### Statistical Analysis

All trials were conducted three times. The *p*-values and kappa values of the RAA and standard PCR assays were calculated. The statistical analysis was conducted with SPSS 21.0 (IBM, Armonk, NY, United States).

## Results

### Primers and Probe Design for the Recombinase-Aided Amplification Assay

**FIGURE 1:**
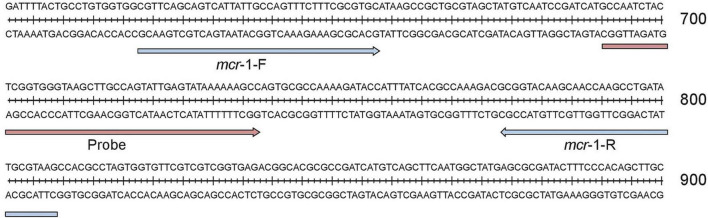
Primer and probe regions in *mcr*-1.

Since the first *mcr*-1 sequence was released in 2015 (NCBI Reference Sequence: NG_050417.1), 31 variants of it have been identified. The genome sequences of all *mcr*-1 genes are almost identical. The primers and probes for this study were manually designed on the specific and conserved region (Figure 1 and Table 2).

### Specificity and Sensitivity Analysis of the Recombinase-Aided Amplification Assay

**FIGURE 2:**
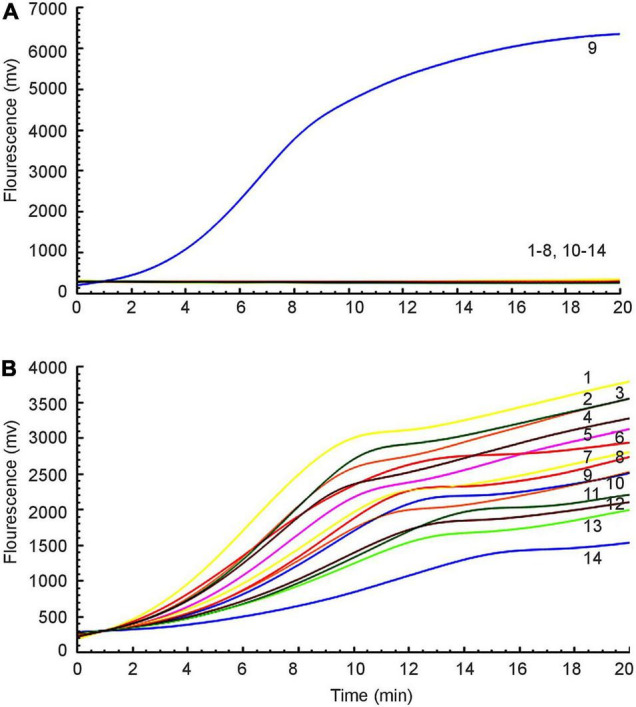
Specificity of the RAA assay. **(A)** Amplification signal was only amplified from *E. coli*^mcr^**^–1^, and no relevant signal was detected in other *mcr*-1-lacking strains. **(B)** All strains produced 16S rRNA gene amplification signals: 1: *P. mirabilis*, 2: *K. pneumoniae* 2146, 3: *K. oxytoca*, 4: *P. aeruginosa*, 5: *S. sonnei*, 6: *A. baumannii*, 7: *C. jejuni*, 8: *E. aerogenes*, 9: *E. coli*^mcr^**^–1^, 10: *S. enteritidis*, 11: *E. cloacae*, 12: *C. freundii*, 13: *P. mirabilis*, 14: *E. coli* ATCC 25922.

Fourteen different strains were used as templates to amplify the *mcr*-1 gene (Table 1). Only from *E. coli*^mcr^**^–1^ did we succeed in detecting amplification signals, while the others were all negative (Figure 2A). As an internal control, the amplification signals of the 16S rRNA were detected from all bacteria (Figure 2B). Therefore, the RAA assay for the detection of *mcr*-1 was 100% specific.

**FIGURE 3:**
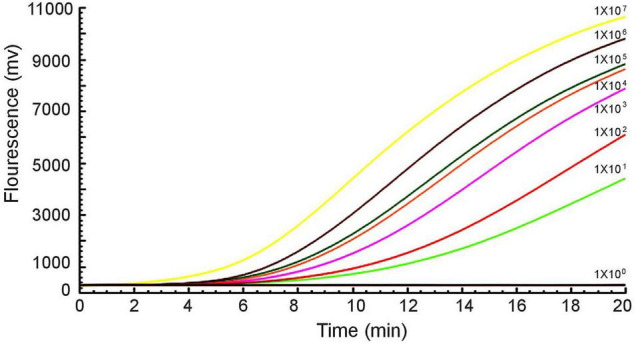
Sensitivity of the RAA assay. An increase in the fluorescence signal was observed from 1 × 10^1^ to 1 × 10^7^ copies/reaction.

Furthermore, a 10-fold gradient dilution series of recombinant plasmids pUC57-*mcr*-1 was used to detect the sensitivity of the RAA assay. As the copies/μL increased from 1 × 10^1^ to 1 × 10^7^, the fluorescence signal increased (Figure 3). The detection limit of the RAA assay was 20 copies per reaction.

### Evaluating the Recombinase-Aided Amplification Assay on Clinical Samples

**FIGURE 4:**
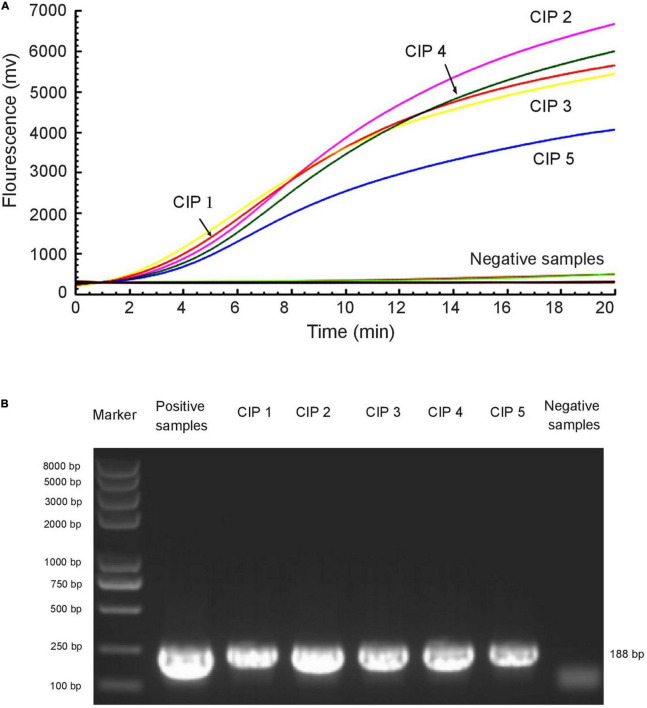
RAA assay was applied to clinical samples. Five *mcr*-1-positive samples were identified among 672 samples by RAA detection **(A)** and standard PCR **(B)**. The *E. coli*^mcr^**^–1^ was used as a positive control and sterile water was used as a negative control.

The RAA assay and the standard PCR assay were simultaneously used to detect *mcr*-1 in 672 samples. Among these, the presence of the *mcr*-1 gene was found in five samples. The two methods gave the same experimental results (Figure 4 and Table 3).

**TABLE 3:** *mcr*-1-Positive Strains isolated from clinical samples.

Isolated strain	Sample ID	RAA	PCR	Sequencing	Transfer efficiency
*E. coli*/*mcr-*1 1	CIP1	+	+	*mcr*-1	3.54 × 10^–2^
*E. coli*/*mcr-*1 2	CIP2	+	+	*mcr*-1	2.06 × 10^–3^
*E. coli*/*mcr-*1 3	CIP3	+	+	*mcr*-1	2.34 × 10^–3^
*E. coli*/*mcr-*1 4	CIP4	+	+	*mcr*-1	0.57
*E. coli*/*mcr-*1 5	CIP5	+	+	*mcr*-1	0.58

*+, mcr-1-positive.*

By screening for colistin resistance, five colistin-resistant *E. coli* were isolated from five clinical samples. All of these *E. coli* had the *mcr*-1 gene, as determined by PCR and sequencing (Table 3). MLST analysis showed that the five *E. coli* belonged to different ST types: *E. coli/mcr*-1 1 (ST21), *E. coli/mcr*-1 2 (ST740), *E. coli/mcr*-1 3 (ST48), *E. coli/mcr*-1 5 (ST809), and *E. coli/mcr*-1 4 (a new ST type).

### Susceptibility Test for the Mobile Colistin Resistance-Positive Bacteria

Following testing with the VITEK^®^ 2 compact system, most of the *mcr*-1-positive *E. coli* were shown to be resistant to colistin, ciprofloxacin, levofloxacin, doxycycline, and trimethoprim, and sensitive to imipenem, amikacin, and meropenem (Table 4). *E. coli/mcr*-1 2 was resistant to ticarcillin/clavulanic acid, piperacillin/tazobactam, and cefoperazone, while the other bacteria were sensitive to these antibiotics. The MICs of colistin and polymyxin B were simultaneously determined by the twofold serial dilution method (Table 4).

**TABLE 4:** Bacterial susceptibilities to antibiotics.

Antibiotic agent	MIC (mg/L)^a^										
	*E. coli/* *mcr*-1 1	*E. coli*/ *mcr*-1 2	*E. coli*/ *mcr*-1 3	*E. coli*/ *mcr*-1 4	*E. coli*/ *mcr*-1 5	*E. coli* J53	*E. coli*-J53 TCp *E. coli*/ *mcr*-1 1	*E. coli*-J53 TCp *E. coli*/ *mcr*-1 2	*E. coli*-J53 TCp *E. coli*/ *mcr*-1 3	*E. coli*-J53 TCp *E. coli*/ *mcr*-1 4	*E. coli*-J53 TCp *E. coli*/ *mcr*-1 5
Ticarcillin/ Clavulanic acid	16^S^	≥ 128^R^	16^S^	≤ 8^S^	16^S^	≤ 8^S^	≤8^S^	≤ 8^S^	≤8^S^	≤ 8^S^	≤8^S^
Piperacillin/ Tazobactam	≤ 4^S^	≥ 128^R^	≤ 4^S^	≤4^S^	≤ 4^S^	≤4^S^	≤ 4^S^	≤4^S^	≤ 4^S^	≤4^S^	≤ 4^S^
Ceftazidime	0.25^S^	32^R^	8^I^	≤ 0.12^S^	32^R^	≤ 0.12^S^	≤0.12^S^	≤ 0.12^S^	≤0.12^S^	≤ 0.12^S^	≤0.12^S^
Cefoperazone	≤ 8^S^	≥ 64^R^	≤ 8^S^	≤8^S^	16^S^	≤ 8^S^	≤8^S^	≤ 8^S^	≤8^S^	≤ 8^S^	≤8^S^
Cefepime	≤ 0.12^S^	≥ 32^R^	8^S*DD*^	≤ 0.12^S^	≥ 32^R^	≤ 0.12^S^	≤0.12^S^	≤ 0.12^S^	≤0.12^S^	≤ 0.12^S^	≤0.12^S^
Aztreonam	≤ 1^S^	≥ 64^R^	16^R^	≤ 1^S^	≥ 64^R^	≤ 1^S^	≤1^S^	≤ 1^S^	≤1^S^	≤ 1^S^	≤1^S^
Imipenem	≤ 0.25^S^	≤0.25^S^	≤ 0.25^S^	≤0.25^S^	≤ 0.25^S^	≤0.25^S^	≤ 0.25^S^	≤0.25^S^	≤ 0.25^S^	≤0.25^S^	≤ 0.25^S^
Meropenem	≤ 0.25^S^	≤0.25^S^	≤ 0.25^S^	≤0.25^S^	≤ 0.25^S^	≤0.25^S^	≤ 0.25^S^	≤0.25^S^	≤ 0.25^S^	≤0.25^S^	≤ 0.25^S^
Amikacin	≤ 2^S^	≤2^S^	≤ 2^S^	≤2^S^	≤ 2^S^	≤2^S^	≤ 2^S^	≤2^S^	≤ 2^S^	≤2^S^	≤ 2^S^
Tobramycin	8^I^	8^I^	≥ 16^R^	≤ 1^S^	8^I^	≤1^S^	≤ 1^S^	≤1^S^	≤ 1^S^	≤1^S^	≤ 1^S^
Ciprofloxacin	≥ 4^R^	≥4^R^	≥ 4^R^	1^R^	2^R^	≤ 0.25^S^	≤0.25^S^	≤ 0.25^S^	≤0.25^S^	≤ 0.25^S^	≤0.25^S^
Levofloxacin	≥ 8^R^	≥8^R^	4^R^	1^I^	4^R^	≤ 0.12^S^	≤0.12^S^	≤ 0.12^S^	≤0.12^S^	≤ 0.12^S^	≤0.12^S^
Doxycycline	≥ 16^R^	≥16^R^	≥ 16^R^	8^I^	≥16^R^	2^S^	2^S^	2^S^	2^S^	2^S^	2^S^
Minocycline	8^I^	≥ 16^R^	4^S^	≤ 1^S^	≥ 16^R^	≤ 1^S^	≤1^S^	≤ 1^S^	≤1^S^	≤ 1^S^	≤1^S^
Tigecycline	1^S^	≤ 0.5^S^	≤0.5^S^	≤ 0.5^S^	≤0.5^S^	≤ 0.5^S^	≤0.5^S^	≤ 0.5^S^	≤0.5^S^	≤ 0.5^S^	≤0.5^S^
Trimethoprim	≥ 320^R^	≥320^R^	≥ 320^R^	≥320^R^	≥ 320^R^	≤ 20^S^	≤20^S^	≤ 20^S^	≤20^S^	≤ 20^S^	≤20^S^
Colistin	4^R^	≥ 16^R^	≥16^R^	8^R^	≥ 16^R^	≤ 0.5^I^	4^R^	4^R^	8^R^	2^I^	2^I^
Colistin^b^	16	16	16	16	16	≤ 0.5	8	8	8	8	8
Polymyxin B^b^	8	8	8	8	8	≤ 0.5	4	4	8	8	8

*S, susceptible; I, intermediate; R, resistance; SDD, susceptible dose-dependent.*

*^a^Unless otherwise indicated, all MICs were determined by VITEK^®^2 system.*

*^b^MIC determined by twofold serial dilution method.*

### Transconjugation of Mobile Colistin Resistance

To investigate the transferability of *mcr*-1, HGT assays were performed using colistin-resistant isolates as donors and sodium azide-resistant *E. coli* J53 as recipient. *mcr*-1 from all five colistin-resistant strains was successfully transferred to *E. coli* J53 recipient strain with some efficiency (Table 3). In contrast to the parental recipient strain, *E. coli* J53 transconjugants showed varying degrees of resistance to colistin and polymyxin B (Table 4). It was not difficult to find that other resistance did not transfer with the *mcr*-1 gene transfer (Table 4). Interestingly, the transfer efficiency of *mcr*-1 from *E. coli/mcr*-1 4 and *E. coli/mcr*-1 5 was much higher than that from other isolated *E. coli* strains, at up to 57–58%. The mechanisms related to this are under investigation.

## Discussion

Polymyxins are a class of medications used in the treatment of systemic infections caused by susceptible strains of multidrug-resistant GNB (Shatri and Tadi, 2022). In GNB, modification of lipid A of LPS is the main mechanism of polymyxin resistance and pEtN transferase enzyme encoded by *mcr*-1 can add a pEtN to the phosphate groups in lipid A (Liu et al., 2016, 2017). *mcr*-1 has been identified in more than 50 countries across the globe, and detected from many different species of GNB (Nang et al., 2019; Xiaomin et al., 2020). Transmission of *mcr*-1 has been observed in all kinds of environment including a variety of water resources, raising the possibility that it could be transferred to humans through HGT (Fernandes et al., 2017; Hembach et al., 2017; Sun et al., 2017). Therefore, an efficient, sensitive and reliable method to detect *mcr*-1 genes is crucial for early diagnosis and infection control in clinical samples.

RAA assay, loop-mediated isothermal amplification (LAMP), and nucleic acid sequence-based amplification (NASBA) are all isothermal amplification techniques, among which RAA assay is the cheapest. As an experimental method with high sensitivity and specificity, RAA assay takes only 20 min to obtain results, while LAMP and real-time PCR take 1–2 h (Du et al., 2021). In addition, RAA assay has been successfully used in identifying SARS-CoV-2, *bla*NDM, *bla*KPC, respiratory syntactical virus, hepatitis B virus, salmonella, and other pathogens (Zhang et al., 2017; Fan et al., 2019; Qi et al., 2019; Shen et al., 2019; Xue et al., 2020a,b). In this study, we developed an RAA assay to detect *mcr*-1 in clinical specimens from children. Among 672 samples, only five of them were *mcr*-1-positive, and the results from RAA assay and standard PCR were identical, indicating that RAA assay is efficient and accurate for detecting the *mcr*-1 gene in clinical samples. The detection rate was close to those in other studies among children (Hu et al., 2017; Wu et al., 2021). Human fecal carriage of *mcr*-1-positive *E. coli* has been detected in many regions (Chan et al., 2018; Zhong et al., 2018); in this study, all *mcr*-1-positive isolated strains were identified as *E. coli*. Five distinct STs detected by MLST showed that *mcr*-1-positive *E. coli* isolates from different origins have high clonal diversity.

HGT of *mcr*-1 is a threat to human health, so it is necessary to calculate the efficiency of *mcr*-1 transfer. The reported transfer efficiency of *mcr*-1 varies greatly depending on the experimental conditions, methods, and strains, ranging from 10^–9^ to 10^–1^ (Anjum et al., 2016; Liu et al., 2016; Quesada et al., 2016; Gutiérrez et al., 2019; Lu et al., 2019; Li et al., 2022). In our study, *mcr*-1 from all five colistin-resistant strains was successfully transferred into the *E. coli* J53 recipient strain by conjugation. Interestingly, the transfer efficiency of *mcr*-1 from *E. coli/mcr*-1 4 and *E. coli/mcr*-1 5 was as high as 57–58%, which is quite high among reported rates. Whether there is a new plasmid that mediates gene transfer will be further studied in the future.

In conclusion, we constructed an RAA assay for *mcr*-1, screened many clinical samples from a pediatric hospital in Beijing, and confirmed the sensitivity, specificity and effectiveness of our method, which will greatly contribute to clinical diagnosis.

## Data Availability Statement

The original contributions presented in the study are included in the article/supplementary material, further inquiries can be directed to the corresponding author/s.

## Ethics Statement

The present project was performed in compliance with the Helsinki Declaration (Ethical Principles for Medical Research Involving Human Subjects) and was approved by the research board of the Ethics Committee of the Capital Institute of Pediatrics, Beijing, China (SHERLLM2022004). All specimens used in this study are part of routine patient management without any additional collection, and all patient data were anonymously reported. Based on the guidelines of the Ethics Committee of the Capital Institute of Pediatrics, no consent was needed in this study.

## Author Contributions

JY and YF designed the study. ZF, YF, CY, NL, RZ, and SL performed the experiments. WX, GX, HZ, and YF collected the clinical samples. ZF, SD, CY, JC, LG, TF, and JF analyzed the results. ZF, YF, and GX wrote the manuscript. JY and GX revised the manuscript. All authors read and approved the final manuscript.

## Conflict of Interest

The authors declare that the research was conducted in the absence of any commercial or financial relationships that could be construed as a potential conflict of interest.

## Publisher’s Note

All claims expressed in this article are solely those of the authors and do not necessarily represent those of their affiliated organizations, or those of the publisher, the editors and the reviewers. Any product that may be evaluated in this article, or claim that may be made by its manufacturer, is not guaranteed or endorsed by the publisher.

## Footnotes

1
https://bigsdb.pasteur.fr/ecoli/primers-used/
